# Thyrotropin receptor antibodies and Graves’ orbitopathy

**DOI:** 10.1007/s40618-020-01380-9

**Published:** 2020-08-04

**Authors:** T. Diana, K. A. Ponto, G. J. Kahaly

**Affiliations:** 1 Molecular Thyroid Research Laboratory, Department of Medicine I (TD, GJK), Johannes Gutenberg University (JGU) Medical Center, 55101 Mainz, Germany; 2 Department of Ophthalmology and Center for Thrombosis and Hemostasis (KAP), Johannes Gutenberg University (JGU) Medical Center, Mainz, Germany

**Keywords:** Graves’ orbitopathy, Thyrotropin receptor, Thyrotropin receptor antibodies, Pathogenesis

## Abstract

**Context and purpose:**

The thyrotropin receptor (TSHR) is the key autoantigen in Graves’ disease (GD) and associated orbitopathy (GO). Antibodies targeting the TSHR (TSHR-Ab) impact the pathogenesis and the course of GO. This review discusses the role and clinical relevance of TSHR-Ab in GO.

**Methods:**

Review of the current and pertinent literature.

**Results:**

GO is the most common extrathyroidal manifestation of GD and is caused by persistent, unregulated stimulation of TSHR-expressing orbital target cells (e.g. fibroblasts and pre-adipocytes). Serum TSHR-Ab and more specifically, the stimulatory Ab (TSAb) are observed in the vast majority of patients with GD and GO. TSHR-Ab are a sensitive serological parameter for the differential diagnosis of GO. TSHR-Ab can be detected either with conventional binding immunoassays that measure binding of Ab to the TSHR or with cell-based bioassays that provide information on their functional activity and potency. Knowledge of the biological activity and not simply the presence or absence of TSHR-Ab has relevant clinical implications e.g. predicting *de-novo* development or exacerbation of pre-existing GO. TSAb are specific biomarkers of GD/GO and responsible for many of its clinical manifestations. TSAb strongly correlate with the clinical activity and clinical severity of GO. Further, the magnitude of TSAb indicates the onset and acuity of sight-threatening GO (optic neuropathy). Baseline serum values of TSAb and especially dilution analysis of TSAb significantly differentiate between thyroidal GD only versus GD + GO.

**Conclusion:**

Measurement of functional TSHR-Ab, especially TSAb, is clinically relevant for the differential diagnosis and management of GO.

## Graves’ orbitopathy and the thyrotropin receptor

**Fig. 1:**
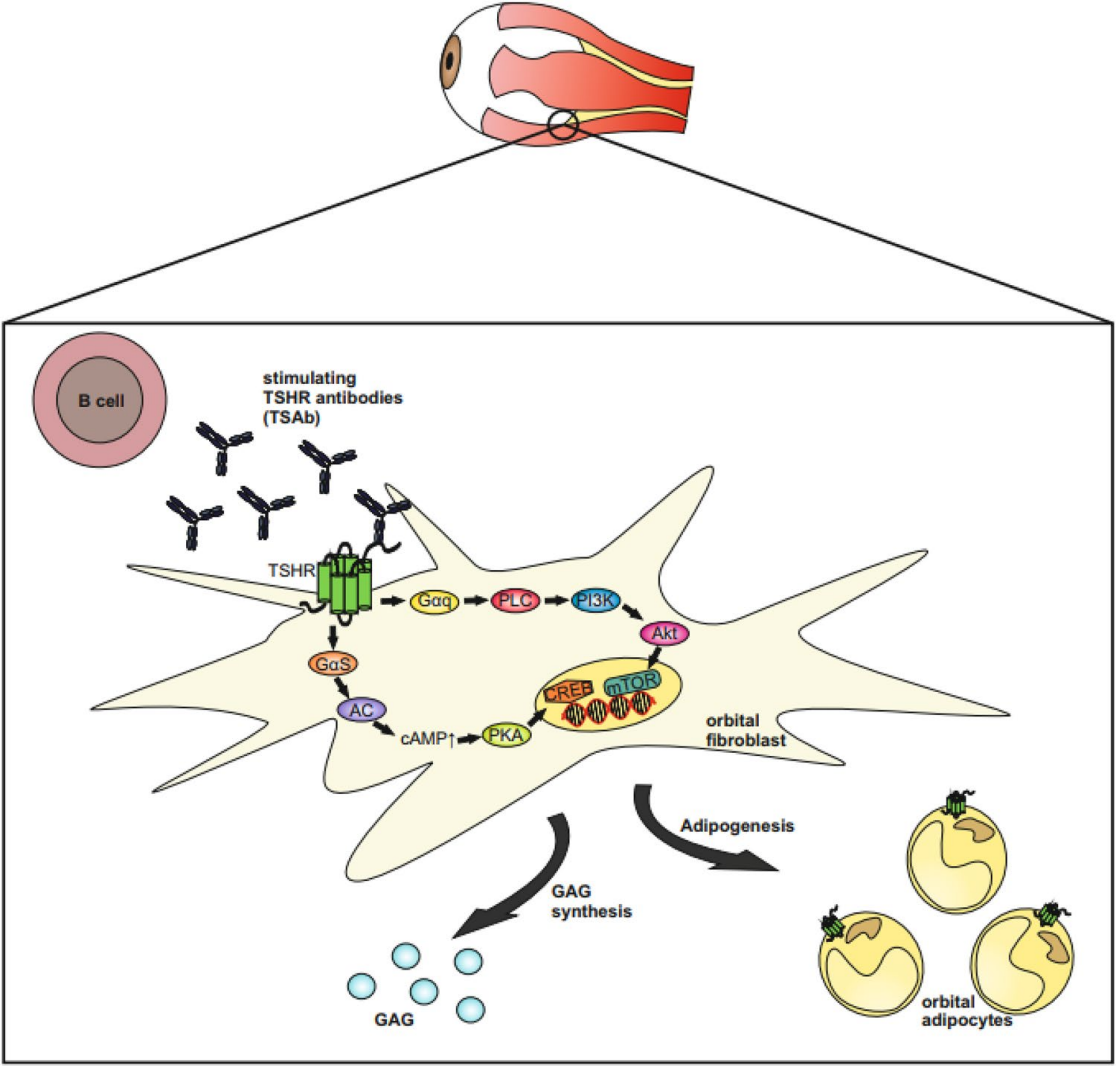
Schematic of the role of TSHR signal transduction pathways in the pathogenesis of GO. The Gα(alpha)s subunit induces the activation of the adenylate cyclase (AC) which causes an increase of cyclic adenosine monophosphate (cAMP) production and further activates the protein kinase A (PKA) and the transcription factor cAMP response element-binding (CREB). The Gαq subunit activates phospholipase C (PLC) which in turn activates the phosphoinositide 3-kinase (PI3K), the protein kinase B (Akt) and the mammalian target of rapamycin (mTOR). Thyrotropin receptor stimulating antibodies (TSAb) bind to orbital fibroblasts and adipocytes in the orbital connective tissue. Binding of TSAb to the TSHR activates orbital fibroblasts hence causing increased synthesis of glycosaminoglycan (GAG) and in the long-term induction of adipogenesis

Graves’ orbitopathy (GO) or thyroid-associated orbitopathy (TAO) is the most common extrathyroidal manifestation of autoimmune Graves’ disease (GD) [1–7]. GO can appear before, during or after onset of the thyroid disease [3, 8, 9]. Asymmetry of GO indicates more severe and active disease as recently shown by a prospective cross-sectional multicenter study of the European Group on Graves’ orbitopathy [10]. Immunohistochemical analysis of orbital tissue provides microscopic alterations and macroscopic manifestations of GO [11]. In patients with GO alterations were observed by overexpression of the TSHR and HLA-DR [11]. Further, orbital fibroblasts were activated by up-regulated proinflammatory and profibrotic various cytokines, adhesion molecules as well as growth factors [11]. Anti-inflammatory and immunosuppressive treatment, e.g. mycophenolate [12], improves GO and is associated with a low rate of mild-to-moderate side effects [13, 14]. The close clinical relationship between Graves’ hyperthyroidism and GO has suggested that immunoreactivity against the thyrotropin receptor (TSHR) present in both the thyroid and orbit underlies both conditions (Fig. 1) [11, 14–16]. A recent animal model of long-term GD was established after repeated immunizations with a vector containing the TSHR [17]. This successful long-term GD model was confirmed by the measurement of functional TSH-R antibodies (Ab) in the animal sera and showed typical morphological changes and glycosaminoglycan deposition in the animal orbits [18]. Further, the levels of TSHR-Ab in subjects with a recent-onset, untreated GO are directly correlated with the clinical activity of the disease, confirming a potential role of these antibodies in the pathogenesis of GO [19]. A prerequisite for involvement of TSHR as an autoantigen in GO is that it be expressed in affected orbital tissues [20]. Several studies demonstrate that TSHR mRNA and protein are present in the orbital tissue of GO patients [11]. Further, TSHR expression has been shown to be higher in GO orbital fat compared with normal orbital adipose tissues. Also, there exists a positive correlation between TSHR mRNA levels in individual GO orbital connective tissue specimens and the patient’s clinical disease activity [21]. The extrathyroidal manifestations of GD, i.e. GO and dermopathy, are due to immunologically mediated activation of TSHR expressing fibroblasts in the extra ocular muscles and skin, proliferation of connective and adipose tissue with accumulation of glycosaminoglycans, leading to the trapping of water and edema [22–29]. Later, fibrosis becomes prominent. The fibroblast activation is caused by pro-inflammatory cytokines derived from locally infiltrating T lymphocytes (CD4+, CD8+, CD45RO+, CD45RB+) [30] and macrophages [31]. Activation of TSHR-expressing orbital fibroblasts by stimulatory TSHR-Ab and pro-inflammatory cytokines causes production of collagen and hydrophilic mucopolysaccharides [28, 32].

The immune response in autoimmune thyroid diseases occurs predominantly within the thyroid gland itself, the site of thyroid antigen expression. A variety of immune cell capture molecules are expressed on the surface of thyroid. Smoking and environmental lifestyle factors, e.g. stress and negative life experiences, also increase the risk for GO [33]. At the cellular level, TSHR-Ab impact oxidative stress caused by increased synthesis of reactive oxygen species (ROS). Indeed, untreated GD/GO has been associated with an increase of several parameters of oxidative stress (i.e. lipid peroxidation) [34]. The TSHR expressed on the plasma membrane of thyroid epithelial cells and orbital target cells, is central to the regulation of thyroid growth and function. However, it is also expressed on a variety of other tissues, including orbital fibroblasts, adipocytes and bone cells. The TSHR is the major autoantigen in the autoimmune hyperthyroidism of GD where T cells and Ab are directed at the TSHR antigen. Stimulatory Ab in GD activate TSHR on thyroid follicular cells, leading to thyroid hyperplasia and unregulated thyroid hormone production and secretion [35]. Stimulatory TSHR-Ab are a hallmark of GD and their production is T cell-dependent and circulating T cells recognize various epitopes of the TSHR.

## Nomenclature

Various terms have been introduced in the literature for the different types of TSHR-Ab [36]. TSHR-Ab, often referred to as TRAb, describes any type of Ab specific to the TSHR, but it is usually applied in reference to Ab measured in an immunoassay. Today, immunoassays are competitive-binding assays that detect TSHR binding inhibitory immunoglobulins (TBII). In contrast, cell-based bioassays measure either TSHR stimulatory antibodies (TSAb), also referred to as TSHR stimulating immunoglobulins (TSI), or TSHR blocking antibodies (TBAb) also referred to as TSHR blocking immunoglobulins (TBI).

## Measurement of thyrotropin-receptor antibodies

TSHR-Ab is the causative agent in GD and the clinical phenotype of GD is induced by prolonged, unregulated stimulation of thyroid cells by stimulating TSHR-Ab that activate the TSHR [37, 38]. TSAb are the only specific biomarkers of GD and GO [7, 39–43] that bind primarily to the large amino-terminal extracellular domain of the TSHR. There are several functional types of TSHR-Ab. TSHR-Ab act either as an agonist, stimulating unregulated thyroid growth and thyroid hormone production, as antagonist, blocking (TBAb) the activity of the natural ligand TSH, or as neutral TSHR-Ab that neither induce nor block the cAMP signal pathway. Neutral TSHR-Ab, however, are able to induce apoptosis, but the clinical significance of this is not clear.

**Fig. 2:**
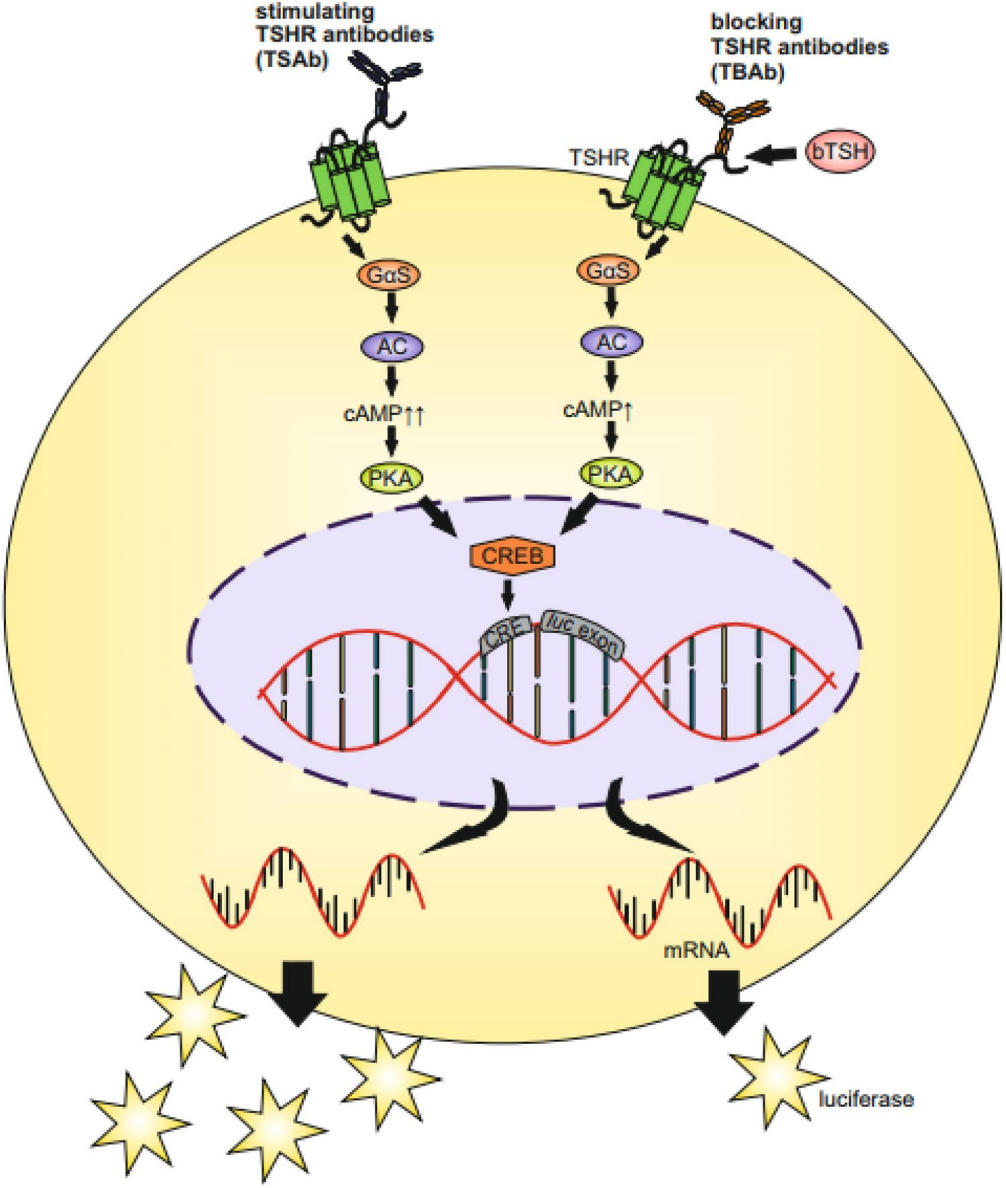
Schematic of functional cell-based bioassays. The Gα(alpha)s subunit induces the activation of the adenylate cyclase (AC) which causes an increase of cyclic adenosine monophosphate (cAMP) production and further activates the protein kinase A (PKA) and the transcription factor cAMP response element-binding (CREB). The Gαq subunit activates phospholipase C (PLC) which in turn activates the phosphoinositide 3-kinase (PI3K), the protein kinase B (Akt) and the mammalian target of rapamycin (mTOR). Serum thyrotropin receptor stimulating antibodies (TSAb) bind to the TSHR which are expressed on the surface of genetically engineered cells. The activation leads to an increase of cyclic adenosine monophosphate (cAMP) production. Subsequently, CREB (cAMP response element-binding) binds to CREs (cAMP response elements) and the luciferase reporter gene (*luc*) is transcribed and translated. Measurement of luciferase activity produces a luminescent signal which can be measured in a luminometer. Serum thyrotropin receptor blocking antibodies (TBAb) inhibit bovine (b) TSH-induced stimulation of luciferase leading to a reduced luminescent signal

The measurement of TSHR-Ab can be performed via competitive-binding immunoassays or with cell-based bioassays (Table 1) [18, 36, 44]. Antibody binding assays only report the presence or absence of TSHR-Ab and their concentration, but do not indicate their functional activity or potency [45–47]. Bioassays, in contrast, indicate whether TSHR-Ab have stimulatory [39, 44, 48] or blocking activity [49, 50] (Fig. 2). Indeed, cell-based bioassays show the functionality of the antibodies that interact with the human TSHR, and can specifically detect TSAb, differentiating between the TSHR antibody types (blocking/stimulating). The TSHR is one of the primary antigens in autoimmune thyroid disease and the critical functional component of bioassays. Bioassays are more sensitive in measuring low serum anti-TSHR-Ab concentrations in comparison to TBII binding assays [44–47]. In the past, bioassays for TSHR-Ab were laborious and complex research tools that required multiple steps and displayed variable sensitivity and specificity. Further, bioassays have been plagued by problems such as poor reproducibility between laboratories and technical problems, i.e. more complex technology and longer processing times.

**Table 1:** Comparison of cell-based bioassays with conventional binding immunoassays for the measurement of thyrotropin receptor autoantibodies (TSHR-Ab)

	Functional bioassays	Binding immunoassays
Differentiation of TSHR-Ab functionality	Discriminate the functional type: Stimulating antibody specific (TSAb) Blocking antibody specific (TBAb)	Do not discriminate the functional type
		Neither specific for TSAb nor TBAb
Measurement	TSAb/TBAb cell-based bioassays measure the sum total of the stimulating and blocking activity of the anti-TSHR activity	Measure total anti-TSHR binding activity with competitive binding immunoassays
	Use of living intact cells	No use of living intact cells
	Transfected cell line: TSHR/reporter gene	Binding immunoassays (TBII) are either automated or ELISA assays
Handling and Performance	Minimal handling of the cells, multiple steps are not required anymore	Easy handling and performance
	Newly developed bioassays require neither IgG purification, nor serum starvation, nor serum concentration	Use of serum
	Requires experienced laboratory technician	
Automation	Not yet	Automation is offered by several manufacturers
Standardization	Standardization of TSAb published, not for TBAb	International standardization
Clinical utility	TSAb measured in a bioassay are a useful biomarker for activity, severity, and/or systemic involvement of Graves’ disease and associated orbitopathy	Lower correlation with clinical activity and clinical severity of Graves’ orbitopathy in comparison to TSAb
	Bioassays are useful for predicting intrauterine and/or neonatal thyroid dysfunction in pregnant women with existing or a history of autoimmune thyroid disease	Low positive and negative predictive values for remission and/or relapse of GD after medical therapy
Availability	FDA-cleared TSAb bioassay offered by several reference laboratories in the US, Europe and Asia	Several assays are commercially available in most labs

However, applications of molecular cloning techniques for the measurement of TSHR-Ab activity have led to significant advances in methodology. This led to the development of clinically useful and improved simplified bioassays by employing various genetically engineered cell lines expressing recombinant TSHR. Currently used methods utilize Chinese hamster ovary (CHO) cells transfected with the cloned TSHR cDNA which are highly sensitive and relatively easy to perform [39, 51–54].

Cell-based bioassays measure the cyclic adenosine 3′, 5′-monophosphate (cAMP) signal transduction mediated by binding of ligand to the TSHR [36, 53]. The activity of IgG is measured on a fully intact functional TSHR holoreceptor expressed on intact living cells in bioassays. Historically bioassays that measure TSAb were based on measurement of cAMP levels in cells using radioimmunoassays (RIA) [41, 42, 55–57]. More recently, cell lines that contain a cAMP-inducible reporter gene such as luciferase have been employed to measure TSAb [39, 44, 48, 58, 59]. Clinically utilized immunoassays are competitive binding assays and are based on the displacement of a tracer, either an anti-TSHR monoclonal antibody (Mab) or previously used radioiodine (RAI) labeled bovine TSH [52, 60–65]. These assays have been referred to as TSHR-binding inhibitory immunoglobulin (TBII) assays [36]. Bioassays that measure TSHR-blocking Ab activity are based on the same cell-based systems, but they detect the ability of patient antisera to block TSH or TSAb stimulated cAMP levels or luciferase expression [49, 50, 66]. During the last ten years functional bioassays as well as TBII binding assays have very much improved and deficiencies in assay standardization has been overcome. Further improvements were achieved by the availability of an international standard (IS), currently the human TSHR-stimulating Mab M22, to replace TSH as calibration material. The second IS (08/204) is available at the National Institute for Biological Standards and Control (NIBSC). The quantitation of one FDA-cleared TSAb bioassay by application of the second IS showed the feasibility of reporting TSAb results in international units (IU) rather than as percentage of specimen-to-reference ratio (SRR%) [48]. The TSAb bioassay demonstrated excellent performance in terms of linear range, limit of quantitation, and precision [48]. Further, the analytical performance of this TSAb bioassay proved to be superior to conventional TBII binding assays [44].

## Clinical application and relevance

### Graves’ thyroidal and extrathyroidal disease

Since the discovery of TSAb as the causative agent of GD several studies have been conducted to use TSAb to monitor the classical treatment of GD with oral antithyroid drugs (ATD) [42, 43]. Since the medical treatment has a response rate of only approximately 50%, differentiation between responders and non-responders to ATD prior to the start of therapy is highly relevant and keenly warranted. Thus, the utility and relevance of the functional TSHR-Ab was demonstrated within a large prospective trial evaluating the predictive role of these Ab during ATD treatment of patients with Graves’ hyperthyroidism [43]. TSAb levels mirrored the severity of GD. Their increase during medical treatment was a marker for ongoing disease activity. TSAb dilution analysis was additionally predictive. In this trial, serum TSAb levels were sensitive, specific, and reproducible biomarkers for GD, reliably predicted response to medical therapy and correlated well with disease severity and extrathyroidal manifestations, i.e. thyroid eye disease [7, 39, 67, 68].

As recently shown [69], TSHR-Ab are the most sensitive serological parameter for the differential diagnosis of GO. However, functional TSHR-Ab, namely TSAb are far better biomarkers for GO. Indeed, in one trial [70], TSAb was the strongest independent predictor of proptosis and extra ocular myopathy (*p* < 0.001). Also, in a cohort of Japanese patients with GO, TSAb levels significantly correlated with GO severity, but no association was found between TBII levels and GO scores [71]. Thus, functional TSAb are associated with clinical activity and clinical severity of GD and GO, and, although it has not been conclusively proven, TSAb most probably cause the extrathyroidal manifestations of GD. TSAb likely bind to and stimulate the activity of the TSHR expressing target cells in the eye, skin and bone, leading to GO, Graves’ dermopathy, and associated acropachy. These effects may be mediated through the TSHR in orbital tissue, and evidence has accumulated that these receptors may be functional [21, 72]. Therefore, the monitoring of TSAb levels [44] add another dimension to the assessment of GD and GO severity in individual patients and the bioassay measures the specific function of Ab that highly correlates with Graves’ activity.

In a cross-sectional study, the relation between TSAb and the onset of dysthyroid optic neuropathy (DON) was evaluated [7]. In 180 patients with autoimmune orbital disease serum TSAb levels were measured using a FDA-cleared bioassay. DON of recent onset or a past history of DON (post-DON) was noted in 30/180 (16.7%) of patients with thyroid eye disease. Nineteen of 20 (96%) patients with DON of recent onset were TSAb positive, and TSAb was found to be associated with DON of recent onset (odds ratio, OR: 20.96; 95% CI 1.064–412.85, *p* = 0.045). Further, TSAb levels correlated with the clinical activity score (*r* = 0.70, *p* < 0.001) and higher levels of TSAb were observed in active versus inactive eye disease (SRR% 485.1 ± 132.3 versus 277.7 ± 143.7, cut-off < 140%; *p* < 0.001). Six of seven (85.7%) patients with inactive eye disease with recent onset DON versus one of four (25%) with active post-DON were TSAb positive (*p* = 0.006). Receiver Operating Characteristic (ROC) analysis revealed a cut-off point for TSAb at 377 SRR% (sensitivity, 0.95; specificity, 0.8). Therefore, according to the above data, TSAb recognize patients with DON of recent onset requiring urgent therapy.

In addition, the clinical relevance of TSAb in GD patients with or without GO was evaluated using a FDA-cleared TSAb bioassay [54]. In GD + GO patients, TSAb was positive in 150/155 (94%) and 6/45 (13%) treated GD patients in the chimeric luciferase reporter gene bioassay. Patients with diplopia, optic neuropathy and smokers were positive for TSAb. Serum TSAb correlated with GO activity (*r* = 0.87, *p* < 0.001) and severity (*r* = 0.87, *p* < 0.001). Both the clinical sensitivity (97% vs. 77%; *p* < 0.001) and specificity (89% vs. 43%, *p* < 0.001) of the TSAb bioassay were higher than TBII in patients with GD + GO. Eleven of 200 (5.5%) TSAb positive and TBII negative patients had GD + GO, while seven of 200 (3.5%) TSAb negative and TBII positive patients had thyroidal GD only.

In a further study using the same bioassay, serum TSAb levels were assessed in 108 untreated patients with GO [5]. The results were compared with a TBII binding assay. Levels of TSAb were detected in 106/108 (98%) patients with GO. All 53 hyperthyroid patients were TSI positive, whereas 47 patients (89%) were binding assay (TBII) positive. All 69 patients with active GO were TSAb positive and only 58/69 (84%) patients were TBII positive. TSAb correlated with both the clinical activity (*r* = 0.83, *p* < 0.001) and clinical severity (*r* = 0.81, *p* < 0.001) of GO. All 59 GO patients with diplopia were TSAb positive and 50/59 (85%) patients were TBII positive. Patients with moderate-to-severe and mild GO, 75/75 (100%) and 31/33 (94%) were TSAb positive compared to TBII positivity in 63/75 (84%) and 24/33 (73%), respectively. Levels of TSAb were higher in moderate-to-severe vs. mild GO (SRR% 489 ± 137 vs. 251 ± 100, *p* < 0.001). Chemosis and GO activity predicted TSAb levels alone (*p* < 0.001). TSAb were higher in patients with chemosis (SRR% 527 ± 131) than in patients without (SRR% 313 ± 127, *p* < 0.001). Thus, the association of TSAb levels with clinical features of GO was much stronger in comparison with TBII.

A controlled, follow-up study was carried out in order to differentiate between sixty patients with thyroidal GD only, GD + GO and healthy controls [73]. Serial dilution analyses of serum were performed and measured with six automated, ELISA, and cell-based assays. In hyperthyroid-untreated thyroidal GD only patients, all undiluted samples were positive with the six assays and revealed negative results at dilution step 1:9 in four of six assays. In contrast, in patients with hyperthyroid-untreated GD + GO patients, all undiluted samples stayed positive up to dilution 1:81 (*p* < 0.001). The positivity rate of serum TSAb in the bioassay for patients with GD + GO was 75%, 35%, 5%, and 0% (all *p* < 0.001) at high dilution steps 1:243, 1:729, 1:2187, and 1:6561, respectively. The five ELISA and/or automated assays verified this obvious difference of anti-TSHR-Ab detection between GD only and GD + GO, noted with the TSAb bioassay; however, the Kronus, Dynex, Cobas, Immulite, and Kryptor assays were all negative in GD only samples at low dilutions of 1:27, 1:9, 1:9, 1:9, and 1:9, respectively. The baseline undiluted samples of GD only vs. GD + GO revealed an overlap in the ranges of TSHR-Ab levels, as measured in the five immunoassays and the bioassay. This significant difference was verified with the five ELISA and/or automated assays in patients with thyroidal GD only and GD + GO. Serum samples from euthyroid GD/GO patients remained TSHR-Ab positive at a dilution of 1:243 after 12-month treatment with methimazole. At dilution step 1:3, the GD samples were already negative. A GD patient with TSHR-Ab positivity at dilution 1:729 developed *de-novo* GO. Therefore, dilution analysis of TSHR-Ab titers significantly enabled the differentiation between the GD phenotypes.

In a further prospective trial the clinical utility of the functional TSHR-Ab was prospectively evaluated in patients with GO [67]. In a total of 101 consecutive patients with severe and active GO ophthalmic, endocrine, and serological investigations were performed. Both, serum levels of TSAb and TBAb were measured with two cell-based luciferase bioassays. All 101 patients with severe and active GO were negative for TBAb. In contrast, 91/101 (90%) patients with GO were TSAb positive. Significant correlations of TSAb were noted with the diplopia score (*p* = 0.016), total severity eye score (*p* = 0.009), proptosis (*p* = 0.007), lid aperture (*p* = 0.003), upper lid retraction (*p* = 0.006), keratopathy (*p* = 0.04), and TBII (*p* < 0.001) and negatively with the duration of GO (*p* = 0.002). Median TSAb values were 418 SRR% (range 28% to 795%). Thus, serum TSAb and not blocking TSHR-Ab (TBAb) are highly prevalent in severe and active GO and correlate with disease severity.

### Pediatric GD/GO

In a large, multicenter cross-sectional study the clinical relevance of TSAb was studied in children with GD, both with GO and without orbital disease [39]. A total of 422 serum samples from 157 children with GD, 101 control individuals with other thyroid and non-thyroid autoimmune diseases, and 50 healthy children were investigated. Serum TSAb levels were measured using a FDA-cleared TSAb bioassay and compared with a TBII binding assay. In 82 untreated children with GD, the sensitivity, specificity, as well as the positive and negative predictive values for TSAb and TBII were 100 and 92.68% (*p* = 0.031), 100 and 100%, 100 and 100% and 100 and 96.15%, respectively. In 157 children with GD, TSAb and TBII were reported in 147 (94%) and 138 (87.9%), (*p* < 0.039), and of the 263 samples in 247 (94%) and 233 (89%), (*p* < 0.0075). In children with GD + GO, TSAb and TBII were observed in 100 and 96% (*p* < 0.001), respectively. Hyperthyroid children with GD + GO revealed significantly higher TSAb levels (*p* < 0.0001) compared to those with thyroidal GD only. No marked differences were observed for TBII between the two groups. Medical treatment after three years (median) showed a decrease of TSAb levels of 69% in thyroidal GD versus 20% in GD + GO (*p* < 0.001). In euthyroid children with GO, all 31 samples were TSAb positive; whereas only 24 were TBII positive (*p* = 0.016). Children with Hashimoto’s thyroiditis, non-autoimmune hyperthyroidism, type 1 diabetes, juvenile arthritis and the healthy controls were all negative for TSAb and TBII. This study demonstrated that TSAb is a sensitive, specific, reliable and reproducible biomarker for pediatric GD/GO and correlates well with disease severity and orbital disease. A further multicenter pediatric study confirmed the above results in European children with GD and GO [74]. Children with GD + GO were TSAb positive in 23/24 (95.8%) samples while children with thyroidal GD only were TSAb positive in 47/53 (88.7%) samples. Levels of TSAb measured with a TSAb bioassay were SRR% 417 ± 135 and 320 ± 157 in children with GD + GO and thyroidal GD only.

### Hashimoto’s thyroiditis and thyroid-associated orbitopathy

Thyroid-associated orbitopathy (TAO) is rare in patients with Hashimoto’s thyroiditis (HT). The prevalence and the predictive risk of TSAb in HT patients with and without TAO were evaluated in a longitudinal observational study encompassing a very large collective of patients with autoimmune thyroiditis and healthy controls [68]. Forty-four of 700 (6%) patients with HT had overt TAO. The highest TSAb levels were detected in patients with active and severe orbitopathy versus those with mild and inactive orbitopathy (median SRR% 486, range 392–592 versus 142, 73–192.5; *p* < 0.001). The OR of TSAb positivity (qualitative OR) for the risk of TAO adjusted for gender and age was 55.9 (95% confidence interval [CI], 24.6–127, *p* < 0.0001), whereas the OR per ten-fold change (quantitative OR) in TSAb SRR% was 133 (95% CI, 45–390, *p* < 0.0001). To the best of our knowledge, this is the first study which confirms the presence of TSAb in HT and associated TAO. Indeed, this study showed that TSAb was strongly associated with orbitopathy in patients with HT, as well as demonstrated the high predictive value of baseline TSAb at the first presentation or patient visit for the development of TAO in patients with HT.

## Conclusion

Functional TSHR-Ab are specific biomarkers for GO and are excellent tools for the accurate management of this complex orbital disease. This short review emphasizes the relevance and clinical utility of measuring functional TSHR-Ab, especially TSAb, for the diagnosis, differential diagnosis, and follow-up of the course of GO and GD.

## Abbreviations

Ab: Antibody
AITD: Autoimmune thyroid disease
ATD: Anti-thyroid drug
ELISA: Enzyme-linked Immunosorbent Assay
GD: Graves’ disease
GO: Graves’ orbitopathy
IgG: Immunoglobulin G
Mab: Monoclonal antibody
SRR%: Percentage of specimen-to-reference ratio
RIA: Radioimmunoassay
ROC: Receiver Operating Characteristic
TAO: Thyroid-associated orbitopathy
TBAb: Thyrotropin-receptor blocking antibodies
TBII: TSHR-binding inhibitory immunoglobulins
TSAb: Thyrotropin-receptor stimulating antibodies
TSH: Thyrotropin
TSHR: Thyrotropin receptor
